# Men’s health policies: long overdue

**DOI:** 10.2471/BLT.24.292925

**Published:** 2025-07-09

**Authors:** Morna Cornell

**Affiliations:** a CIDER (Centre for Integrated Data and Epidemiological Research), Faculty of Health Sciences, University of Cape Town, Anzio Rd, Observatory, Cape Town 7925, South Africa.

In adopting *Transforming our world: the 2030 Agenda for Sustainable Development*, countries pledged to reach the furthest behind first. However, to achieve this goal, major gender disparities in health-care access and outcomes must be addressed. Globally, men have shorter life expectancy and higher mortality than women. In 2023, global life expectancy at birth was 71 years for men and 76 for women, a gap that has been consistent since 1950.^1^ The global mortality rate in 2023 was 176 deaths/1000 for men and 113 deaths/1000 for women.^2^

These gender disparities in health are more pronounced in groups of men who are marginalized by race, disability, age or sexual orientation, particularly in post-colonial societies. For example, in southern Africa, the epicentre of the human immunodeficiency virus and tuberculosis epidemics, the migrant labour system disempowered and negatively affected the health of millions of African men and their families. Young men were placed in urban environments with extremely poor living and working conditions. These workers then returned to rural areas, spreading new diseases within rural populations.^3^

Yet responses to gender inequities affecting men’s health have been uneven and frequently polarized. Historically, men’s health has largely been ignored by international health agencies, funders and national programmes,^4^^–^^6^ a situation that has been described as a systematic neglect.^7^ But targeting men’s health is politically sensitive: these discussions have been, and sometimes still are, perceived as reinforcing male privilege, rather than realizing men’s right to health. Men are still not identified as a vulnerable group in most national health policies. Men and women are treated as competing populations, with men somehow less deserving of attention. Yet, given the evidence on health access and outcomes, men are clearly the furthest behind.^5^^,^^8^

Over the past 15 years, a small number of committed individuals and organizations have focused attention on and advocated for men’s health.^9^^,^^10^ As a result, a few countries have formulated men’s health policies, in some cases up to 20 years after formulating policies for women, older people and people with disabilities. The Irish Department of Health and Children led the way with a comprehensive report on men and their health. Building on this report, Australia, Brazil, Islamic Republic of Iran, Malaysia, Mongolia, South Africa, the World Health Organization (WHO) European Region and the Quebec province in Canada have since produced policies addressing men’s health. 

These policies confirm that men have higher mortality and lower engagement with health services than women, and address individual and systemic risk factors driving these disparities. Policies propose entry points to engage men in care including fatherhood in Brazil, voluntary medical male circumcision in South Africa and an approach in Quebec that promotes positive aspects of masculinity. In Australia and Ireland, the policies prioritize the health needs of groups of marginalized men. In Leeds, United Kingdom of Great Britain and Northern Ireland, gender is now considered when commissioning all new services, which has changed the way in which interventions such as cancer and suicide risk prevention are provided. Gender is integrated into broader development planning in Mongolia, while policy-makers in the Islamic Republic of Iran have identified context-specific risk factors for men’s health. In a concise overview of key issues, the WHO Regional Office for Europe highlights men’s high burden of injury, road traffic accidents, suicide and mental health, and shows how financial insecurity and migration increase men’s vulnerability and social exclusion.

However, most policies have insufficient focus on monitoring and lack specific, achievable targets and timelines. Existing country policies would be strengthened by clear monitoring and evaluation frameworks and implementation plans. To date, only the Irish policy has undergone a formal evaluation,^11^ which showed a considerable impact on community-level interventions, research and training for health and related professionals. However, the evaluation found that the recommendations and scope were too extensive, undermining the policy’s impact, and success in addressing broader structural risk factors was limited.

For men, one of the major structural risk factors is violence, fuelled by alcohol and firearms. Globally, the gender disparity in mortality is most evident in deaths from interpersonal violence, especially among young, marginalized men. For example, in 2017, South Africa had seven times the global homicide rate, with no decrease since 2009.^12^ Homicide victims were predominantly male (87%; 16 835/19 477) and young (aged 15–44 years). Alcohol use was strongly associated with violent deaths, and one third of all homicides were firearm-related. Stricter legislation can reduce major risk factors, but strong global alliances are needed to challenge the powerful alcohol and firearm lobbies and reduce the levels of interpersonal violence.

To improve men’s health, the global health community needs to be more ambitious in proposing systemic changes. Future policies require an increased focus on the social determinants of health: the conditions under which people live, work and age, and the inequities that give rise to these conditions. Even in a high-income country like the United Kingdom of Great Britain and Northern Ireland, for example, there is a 27-year difference in life expectancy for different groups of men depending on socioeconomic status, far larger than the gap between men and women.^9^

In addition to national policies, global health institutions and funding agencies have an enormous influence on health responses, and many continue to explicitly equate gender with women, such as the United Nations (UN) Inter-Agency Network on Women and Gender Equality, the Joint United Nations Programme on HIV/AIDS (UNAIDS) *Agenda for accelerated country action for women, girls, gender equality and HIV: operational plan* and the sustainable development goal (SDG) 5 on achieving gender equality and empowering all women and girls. A review of 37 regional and global health policy documents on sexual and reproductive health found that only five of them included targets for men.^13^ When men were included in global health policy documents, it was frequently in an instrumental way, to protect the health of women, rather than to address the health needs of men. Language in global goals should be reviewed for consistency across four critical, interlinked goals – poverty, health, gender equality and reducing inequality – to ensure that the SDGs are evidence-based and truly equity-focused.

In recent years, some positive developments have occurred: some governments have adopted men’s health policies; men’s health organizations have emerged in many countries and globally; a growing body of research has been published in dedicated men’s health journals; UNAIDS and WHO have shown an increasing interest in this topic; and evidence of good practices is emerging. Robustly evaluated projects now exist, demonstrating that a gender-targeted approach works, described in the Global Action on Men’s Health *Delivering men’s health* report.^14^ Equally important is the inclusion of men in other health policies such those related to cancer, sexual health, obesity and mental health. For example, the national suicide strategy of the United Kingdom focuses on men, while the European Commission recommends introducing prostate cancer screening programmes across the European Union (EU) countries. In another positive development, over 40 countries have introduced human papillomavirus vaccination for girls and boys, a measure the European Commission recommends for EU Member States.

These developments are encouraging but more action is needed. As governments work towards universal health coverage, men’s health must be recognized as a fundamental issue, not a peripheral consideration. Addressing gender inequities in health, including those affecting men, is essential to ensuring that no one is left behind. 

## References

[1] Life expectancy at birth, total (years) 1960-2023. World Bank Open Data. Washington, DC: World Bank; 2024. Available from: https://data.worldbank.org/indicator/SP.DYN.LE00.IN?view=chart [cited 2025 Jun 17].

[2] Death rate, crude (per 1,000 people), 1960-2023. World Bank Open Data. Washington, DC: World Bank; 2024. Available from: https://data.worldbank.org/indicator/SP.DYN.CDRT.IN?view=chart [cited 2025 Jun 17].

[3] Lurie MN, Williams BG. Migration and Health in Southern Africa: 100 years and still circulating. Health Psychol Behav Med. 2014 Jan 1;2(1):34–40. 10.1080/21642850.2013.866898 24653964 PMC3956074

[4] Cornell M, McIntyre J, Myer L. Men and antiretroviral therapy in Africa: our blind spot. Trop Med Int Health. 2011 Jul;16(7):828–9. 10.1111/j.1365-3156.2011.02767.x 21418449 PMC3749374

[5] Hawkes S, Buse K. Gender and global health: evidence, policy, and inconvenient truths. Lancet. 2013 May 18;381(9879):1783–7. 10.1016/S0140-6736(13)60253-6 23683645

[6] No man left behind. Lancet Glob Health. 2020 Dec;8(12):e1444. 10.1016/S2214-109X(20)30490-3 33220202

[7] Horton KC, White RG, Houben RMGJ. Systematic neglect of men as a key population in tuberculosis. Tuberculosis (Edinb). 2018 Dec;113:249–53. 10.1016/j.tube.2018.09.006 30287201

[8] Leon N, Colvin C. Gone missing: the treatment of men in global cancer policy. London: Global Action on Men’s Health; 2023. Available from: https://gamh.org/wp-content/uploads/2023/06/GAMH-Men-and-Cancer-Policy-Report.June-2023.Final_.HighRes.pdf [cited 2025 Jun 19].

[9] White A, Tod M. The need for a strategy on men’s health. Trends Urol Men’s Health. 2022;13(2):2–8. 10.1002/tre.842

[10] Baker P. Worth the paper they’re written on? The potential role of national men’s health policies. Eurohealth (Lond). 2015;21(4):27–9.

[11] Baker P. Review of the National Men’s Health policy and Action Plan 2008-2013. Final report for the Health Service Executive. Dublin: Department of Health and Children; 2015. Available from: https://gamh.org/wp-content/uploads/2015/07/Ireland-Mens-Health-Policy-Review.Final-Full-Report.2015.pdf [cited 2025 Jun 19].

[12] Matzopoulos R, Prinsloo MR, Mhlongo S, Marineau L, Cornell M, Bowman B, et al. South Africa’s male homicide epidemic hiding in plain sight: Exploring sex differences and patterns in homicide risk in a retrospective descriptive study of postmortem investigations. PLOS Glob Public Health. 2023 Nov 22;3(11):e0002595. 10.1371/journal.pgph.0002595 37992033 PMC10664949

[13] Shand T, Evoy C. Out of focus: the representation of men in regional and global sexual and reproductive health policy. London: Global Action on Men’s Health; 2024. Available from: https://gamh.org/wp-content/uploads/2024/08/Sexual_Health_Report_SEPT24FINAL.pdf [cited 2025 Jun 19].

[14] Delivering men’s health. London: Global Action on Men’s Health; 2021. Available from: https://gamh.org/wp-content/uploads/2021/09/Delivering-Mens-Health-report.pdf [cited 2025 Jun 18].

